# Infliximab in ankylosing spondylitis: alone or in combination with methotrexate? A pharmacokinetic comparative study

**DOI:** 10.1186/ar3350

**Published:** 2011-06-03

**Authors:** Denis Mulleman, Francine Lauféron, Daniel Wendling, David Ternant, Emilie Ducourau, Gilles Paintaud, Philippe Goupille

**Affiliations:** 1 Université François-Rabelais de Tours, Centre National de la Recherche Scientifique UMR 6239 GICC (Génétique Immunothérapie Chimie et Cancer), 3 rue des Tanneurs, F-37041 Tours Cedex 1, France; 2 Service de Rhumatologie, Centre Hospitalier Régional et Universitaire de Tours, avenue de la République, F-37044 Tours Cedex 9, France; 3 Université de Franche-Comté, EA 4266 API (Agents Pathogènes et Inflammation), Hôpital Saint Jacques, 2 place Saint-Jacques, F-25030 Besançon Cedex, Besançon, France; 4 Service de Rhumatologie, Centre Hospitalier Régional et Universitaire de Besançon, Hôpital Jean Minjoz, 3 boulevard Alexander Fleming, F-25030 Besançon Cedex, Besançon, France; 5 Laboratoire de Pharmacologie-Toxicologie, Centre Hospitalier Régional et Universitaire de Tours, 2 boulevard Tonnellé, F-37044 Tours Cedex 9, France; 6 Institut National de la Santé et de la Recherche Médicale CIC 202, 2 boulevard Tonnellé, F-37044 Tours Cedex 9, Tours, France

## Abstract

**Introduction:**

Methotrexate (MTX) has been shown to modify infliximab pharmacokinetics in rheumatoid arthritis. However, its combination with infliximab in the treatment of ankylosing spondylitis (AS) is not recommended. The objective of this study was to examine the influence of MTX on infliximab exposure in patients with AS.

**Methods:**

Patients with AS patients who had predominantly axial symptoms were randomised to receive infliximab alone (infusions of 5 mg/kg at weeks 0, 2, 6, 12 and 18) or infliximab combined with MTX (10 mg/week). Infliximab concentrations were measured before and 2 hours after each infusion and at 1, 3, 4, 5, 8, 10, 14 and 18 weeks. We estimated individual cumulative area under the concentration versus time curves (AUC) for infliximab concentration between baseline and week 18 (AUC_0-18_). Clinical and laboratory evaluations were performed at each visit. The Bath Ankylosing Spondylitis Disease Activity Index (BASDAI) score was the primary end point for clinical response.

**Results:**

Twenty-six patients were included (infliximab group: *n *= 12, infliximab + MTX group: *n *= 14), and 507 serum samples were available for measurement of infliximab concentration. The two groups did not differ with regard to AUC_0-18 _or evolution of BASDAI scores and biomarkers of inflammation.

**Conclusions:**

The combination of MTX and infliximab does not increase the exposure to infliximab over infliximab alone in patients with AS.

**Trial registration:**

ClinicalTrials.gov: NCT00507403

## Introduction

Infliximab, a chimeric monoclonal antibody to TNF-α, showed efficacy for ankylosing spondylitis (AS) in a randomised, placebo-controlled trial in which 61.2% of the patients were responders at 24 weeks [1]. Although methotrexate (MTX) is often used for patients with predominantly peripheral AS and those with psoriatic arthritis, the few attempts to treat predominantly axial disease were disappointing. Haibel *et al*. [2] studied 20 patients with AS who received MTX 15 to 20 mg/week subcutaneously and found no difference in Assessment in Ankylosing Spondylitis 20% improvement criteria (ASAS 20) scores before and 16 weeks after treatment. Until now, MTX has been evaluated in only three small, randomised, controlled trials [3-5], and a Cochrane review [6] concluded that there was insufficient evidence to support the use of MTX for AS with predominantly axial symptoms.

Data comparing infliximab with and without MTX treatment in AS are sparse and conflicting. Pérez-Guijo *et al*. [7] found a greater reduction in Bath Ankylosing Spondylitis Disease Activity Index (BASDAI) scores with infliximab + MTX treatment than with infliximab alone, whereas Breban *et al*. [8] found no statistically significant difference between patients who did or did not receive MTX in a subset of AS patients receiving treatment with infliximab by an on-demand strategy. However, in the latter study, patients receiving MTX showed a better response and fewer reactions to infusions than did patients not receiving MTX, although the results were not statistically significant [8]. Currently, regarding TNF-α antagonist therapy for patients with AS or psoriatic arthritis, the French Society for Rheumatology recommendations suggest that there is insufficient evidence for concomitant disease-modifying antirheumatic drugs improving the effectiveness of TNF-α antagonist therapy [9].

To date, no study has used infliximab exposure as an end point to compare treatment with the combination of infliximab and MTX with infliximab alone in AS with predominantly axial symptoms. Indeed, if such a combination increases exposure to infliximab, it should improve response and may be recommended in clinical practice. In the present study, we compared the individual exposure to infliximab of AS patients with predominantly axial symptoms receiving infliximab alone or infliximab and MTX combined.

## Materials and methods

### Patients and study protocol

From January 2008 to April 2009, AS patients with predominantly axial symptoms were recruited to participate in this two-centre, open-label, prospective, randomised study comparing treatment with infliximab alone and infliximab with MTX. All patients fulfilled the New York revised criteria for AS [10]. Infliximab was given intravenously (5 mg/kg) at weeks 0, 2, 6, 12 and 18 in accordance with our guidelines [9]. MTX 10 mg was given orally every week. After patients were randomised to a treatment group, a total of 12 visits were scheduled at each infliximab infusion and between infusions at 1, 3, 4, 5, 8, 10 and 14 weeks. Blood samples were collected before and two hours after the end of each infusion and at each visit. We estimated that we needed about 30 patients to compare infliximab exposure between the two treatment groups. The study protocol was in compliance with the Declaration of Helsinki, approved by the ethic committee of Tours University Hospital and registered (ClinicalTrials.gov ID: NCT00507403). All patients gave their informed consent to participate in the study.

### Clinical measurements

At each visit, patients were asked to complete a BASDAI questionnaire and were classified as responders if their BASDAI score (on a 10-point scale) at week 18 was two points lower than at baseline [9,11]. Treatment response was also assessed according to the Assessment in Ankylosing Spondylitis 20% improvement criteria (ASAS 20).

### Serum infliximab and antibodies toward infliximab concentrations

Analyses of serum infliximab and antibody toward infliximab (ATI) concentrations were centralised in Tours University Hospital. Infliximab serum concentration was measured in samples by using ELISA as described previously [12]. Serum concentration of ATI was measured by using a double-antigen ELISA on the basis of capture by infliximab-coated microplates and detection by peroxidase-conjugated infliximab. This assay was standardised by the use of a mouse monoclonal antibody against human immunoglobulin G. The positive threshold of detection was 0.07 mg/L. Because of the interference of circulating infliximab, only sera with infliximab concentrations < 2 mg/L were tested.

### Statistical analysis

Individual exposure to infliximab was estimated by the cumulative area under the concentration versus time curve between baseline and week 18 (AUC_0-18_) by population pharmacokinetics modelling. The best description of infliximab concentrations was obtained by use of a two-compartment model with first-order distribution and elimination constants. Characteristics at baseline and the proportion of responders in each group were compared by using nonparametric tests. The results are presented in Table 1 as medians [range] unless otherwise stated. Statistical analysis involved the use of R 2.7.2 software [13].

**Table 1:** Baseline characteristics of 26 patients with ankylosing spondylitis and predominantly axial symptoms randomised to receive infliximab or infliximab + methotrexate^a^

Characteristics	Infliximab (*n *= 12)	Infliximab + MTX (*n *= 14)	*P *value
Sex, men/women (*n*/%)	9/3 (75/25)	11/3 (79/21)	0.8
Median age, years	42.5 [27 to 59]	45.5 [29 to 55]	0.3
Median disease duration, years	4.0 [0 to 28]	4.5 [1 to 19]	0.9
Human leukocyte antigen B27^+^, *n *(%)	9 (75)	10 (71)	0.8
NSAIDs, *n *(%)	8 (67)	12 (86)	0.5
Previous TNF-α, *n *(%)	2 (17)	2 (14)	1.0
Median BASDAI score	5.8 [3.9 to 8.4]	7.0 [5.0 to 8.2]	0.2
Median C-reactive protein, mg/L	3.6 [0.5 to 18.0]	2.7 [0.5 to 31.2]	0.5

^a ^MTX: methotrexate; BASDAI: Bath Ankylosing Spondylitis Disease Activity Index; NSAIDs: nonsteroidal anti-inflammatory drugs. Results are given as medians [range] unless otherwise indicated.

## Results

**Figure 1:**
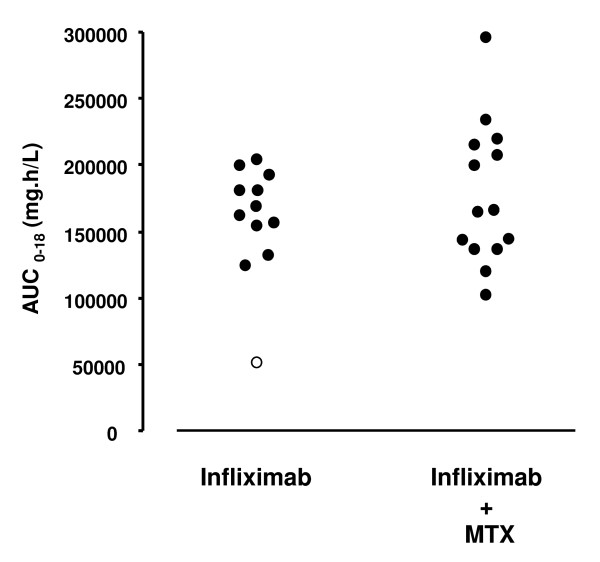
**Area under the concentration curve versus the time curve for serum infliximab concentration between baseline and week 18 (AUC_0-18_) for patients with ankylosing spondylitis who had predominantly axial symptoms and were receiving infliximab or infliximab + methotrexate (MTX) treatment**. The patient with anti-infliximab antibodies is shown as an open circle.

We included 26 patients: 12 in the infliximab-only group and 14 in the infliximab + MTX group. The patients' characteristics at baseline are given in Table 1. A total of 507 samples were available for measurement of serum infliximab concentrations. Individual AUC_0-18 _results for infliximab are shown in Figure 1. The two treatment groups did not differ in terms of AUC_0-18 _results. The median AUC_0-18 _value was 165,502 mg/hour/L [50,569 to 203,782] for the infliximab-only group and 164,222 mg/hour/L [102,165 to 295,858] for the infliximab + MTX group. Likewise, MTX had no effect on estimated pharmacokinetics parameters (data not shown). Positivity for ATI was detected in only one patient, a 43-year-old woman in the infliximab-only group who was negative for human leukocyte antigen B27. At weeks 4, 5 and 6, her infliximab concentrations were < 1 mg/L and ATIs were detected at weeks 12 and 18. Her exposure to infliximab was low (AUC_0-18 _= 50,569 mg/hour/L), and the infliximab elimination clearance was twice that of ATI-negative patients (data not shown).

The two groups did not differ with regard to clinical activity or inflammation. Responses between the two groups were similar at week 18 (Additional file 1). Responders displayed a rapid decrease in BASDAI score compared with nonresponders, with a statistically significant difference observed at week 6 and thereafter (Additional file 2). The proportions of previous anti-TNF-α and current NSAIDs were similar in both responders and nonresponders.

## Discussion

We did not observe any influence of MTX on infliximab exposure in AS patients with predominantly axial symptoms. Our study is the first to answer the question whether MTX may affect infliximab pharmacokinetics in AS with predominantly axial symptoms. Previously, Krisiek *et al*. [14] analysed blood samples from AS patients receiving treatment with infliximab by an on-demand strategy with or without MTX. In their *post hoc *analysis involving 34 patients receiving MTX and 42 patients not receiving MTX, infliximab concentrations were measured at weeks 2 and 6 after treatment. The authors found no significant difference in infliximab levels between the two groups. Our results are in agreement with these previously published results and provide a precise estimation of the drug exposure with repeated and prolonged measurement of infliximab.

We found no difference between treatment groups with regard to change in BASDAI scores or ASAS 20 criteria, findings which are in agreement with results from previous controlled trials [3-5]. Although clinical outcome was not our objective, our results do not argue for a clinical benefit of adding MTX to infliximab treatment (5 mg/kg/infusion) for AS with predominantly axial symptoms. Patients who were responders at week 18 showed a rapid decrease in BASDAI scores, which suggests that an early decrease in BASDAI score may predict a response later on. By contrast, a patient who has not responded at 6 weeks after initiation seems likely to be a primary nonresponder.

The MTX dosage we used, 10 mg/week orally, was in agreement with the recommendation by an expert opinion of a broad international panel of rheumatologists [15]. A subcutaneous route or higher dosage than 10 mg/week would not likely have changed our results, because a previous study found no clinical response in patients with AS receiving MTX 15 to 20 mg/week subcutaneously [2].

We observed an important variability in infliximab exposure in both groups of patients. This variability has been reported in previous studies in which trough concentration was measured [14,16]. Our study provides a more precise characterisation of this variability and confirms that exposure to treatment differs considerably among individuals.

We found that one patient was positive for ATI. Of note, this patient did not receive MTX, and this finding raises the question of the hypothetical role of immunosuppressive agents in preventing immunisation as reported in rheumatoid arthritis (RA) and Crohn's disease [17-19]. Because of the small number of participants and the relatively short duration of the study, no conclusion can be drawn regarding the potential role of MTX in reducing immunogenicity in AS.

Our results differ from those of Maini *et al*. [17], who found that MTX increased infliximab concentrations in RA. In RA, disease activity at baseline was found to be negatively associated with infliximab trough concentration at 6 weeks after initiation of treatment [20,21]. This phenomenon may be explained by targeted mediated-drug disposition and high levels of TNF-α (which corresponds to antigenic burden) binding infliximab, which influence its pharmacokinetics. MTX is effective as monotherapy in patients with RA by decreasing TNF-α levels, which in turn increase infliximab concentrations [17]. That MTX did not improve infliximab exposure in our study patients with AS who had predominantly axial symptoms may reflect the fact that TNF-α antigenic burden is lower in AS than in RA.

## Conclusion

MTX seems to have no significant effect on infliximab pharmacokinetics in AS with predominantly axial symptoms. These results do not argue for adding MTX to infliximab therapy in this condition.

## Abbreviations

AS: ankylosing spondylitis; ASAS 20: Assessment in Ankylosing Spondylitis 20% improvement criteria; ATI: antibody toward infliximab; AUC: area under the concentration versus time curve; BASDAI: Bath Ankylosing Spondylitis Disease Activity Index; ELISA: enzyme-linked immunosorbent assay; MTX: methotrexate; NSAID: nonsteroidal anti-inflammatory drug; RA: rheumatoid arthritis; TNF-α: tumour necrosis factor α.

## Competing interests

DM has a consultancy with Schering-Plough. Payment for lectures was received from Union Chimique Belge, Merck Sharpe & Dohme, Roche and Amgen. Travel, accommodations and meeting expenses were paid by Roche, Bristol-Myers Squibb, Servier and Abbott Laboratories. FL received compensation for travel, accommodations and meeting expenses from Abbott Laboratories and Servier. DW is a board member for Pfizer; has grants or grants pending from Abbott Laboratories, BMS, Schering-Plough, Wyeth, Roche-Chugai and Servier; has received payment for lectures from Abbott Laboratories, BMS Schering-Plough, Pfizer and Roche Chugai; and has received compensation for travel, accommodations and meeting expenses from Abbott Laboratories, BMS and Amgen. DT has no competing interests to declare. ED has a consultancy at Abbott Laboratories and received compensation for travel, accommodations and meeting expenses from UCB, Servier and Roche. GP is a board member for Roche and holds a consultancy with LFB (Laboratoire Français du fractionnement et des biotechnologies), received payment for the development of educational presentations from Janssen-Cilag and received compensation for travel, accommodations and meeting expenses from Roche. PG is a board member of and has consultancies with Abbott Laboratories, BMS and Pfizer; received payment for lectures from Abbott Laboratories, BMS, MSD, Pfizer, Roche and Schering-Plough; and was compensated for travel, accommodations and meeting expenses by Abbott Laboratories, MSD, Pfizer, Roche and Schering-Plough.

## Authors' contributions

DM drafted the manuscript. FL and ED participated in patient clinical assessment and performed the statistical analysis. DW participated in patient clinical assessment and helped draft the manuscript. PG supervised the study design and helped draft the manuscript. DT performed the statistical analysis and pharmacokinetics modelling and helped draft the manuscript. GP participated in the study design and helped draft the manuscript. All authors read and approved the final manuscript.

## Supplementary Material

Additional file 1 **Figure S1**. Percentage of responders according to Bath Ankylosing Spondylitis Disease Activity Index (BASDAI) score ( > 2-point reduction between baseline and week 18) and Assessment in Ankylosing Spondylitis 20% improvement criteria (ASAS 20) for patients with ankylosing spondylitis who had predominantly axial symptoms and were receiving infliximab or infliximab + methotrexate (MTX) treatment. Click here for file

Additional file 2 **Figure S2**. Changes in BASDAI scores for treatment responders (filled circles; *n *= 12) and nonresponders (open circles; *n *= 14) receiving infliximab alone or infliximab + methotrexate treatment. ^†^*P *< 0.05. ^‡^*P *< 0.01. Click here for file
