# Supplementary figures and images for: Infliximab in ankylosing spondylitis: alone or in combination with methotrexate? A pharmacokinetic comparative study
Source: Arthritis Res Ther. 2011 Jun 3;13(3):R82. doi: 10.1186/ar3350 (PMC3218893; doi:10.1186/ar3350)

## Slide 1
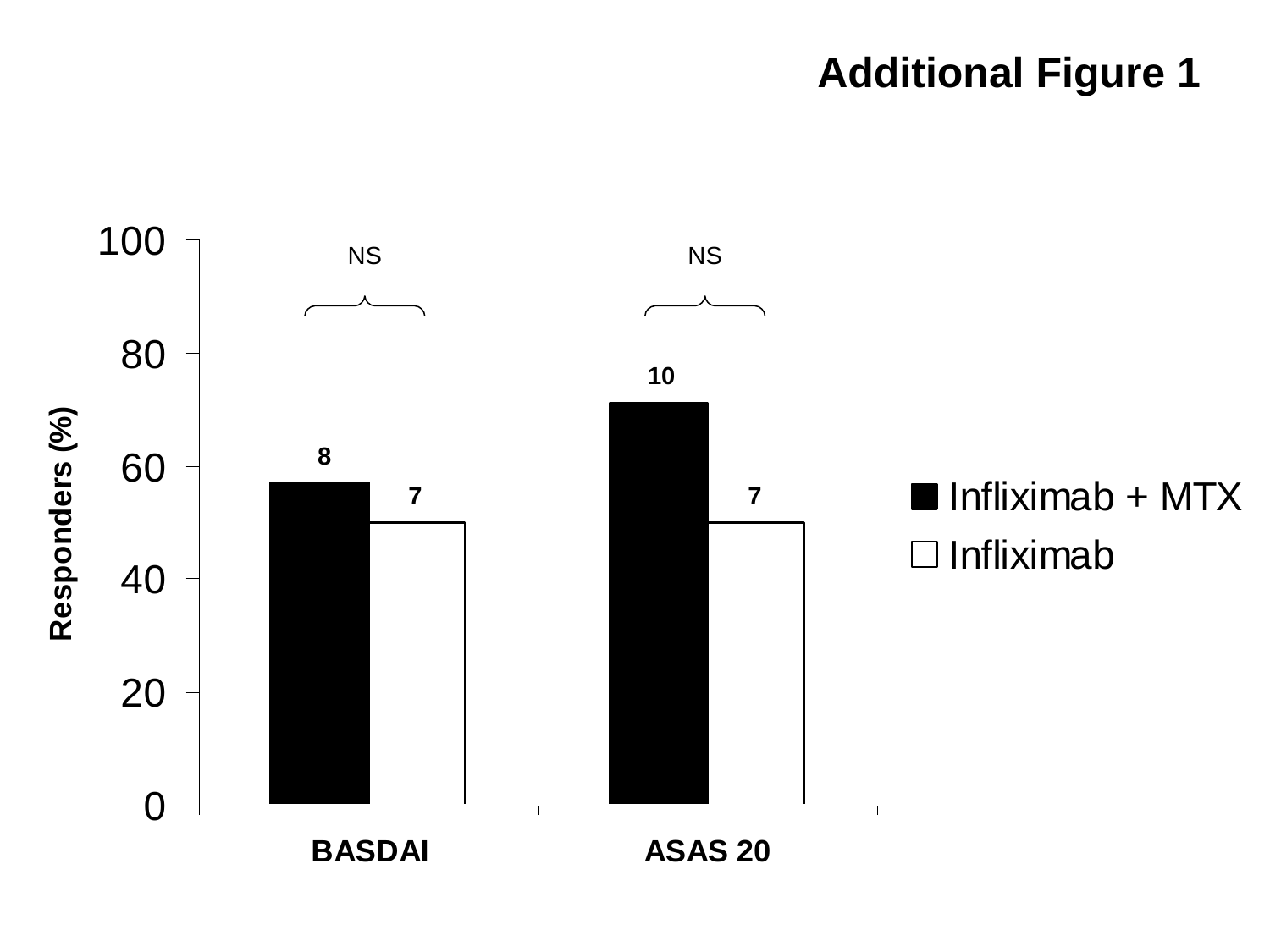

Additional Figure 1
NS
NS
10
8
7
7

Supplement: Additional file 1 — Figure S1. Percentage of responders according to Bath Ankylosing Spondylitis Disease Activity Index (BASDAI) score ( > 2-point reduction between baseline and week 18) and Assessment in Ankylosing Spondylitis 20% improvement criteria (ASAS 20) for patients with ankylosing spondylitis who had predominantly axial symptoms and were receiving infliximab or infliximab + methotrexate (MTX) treatment. [file ar3350-S1.PPT]

## Slide 1
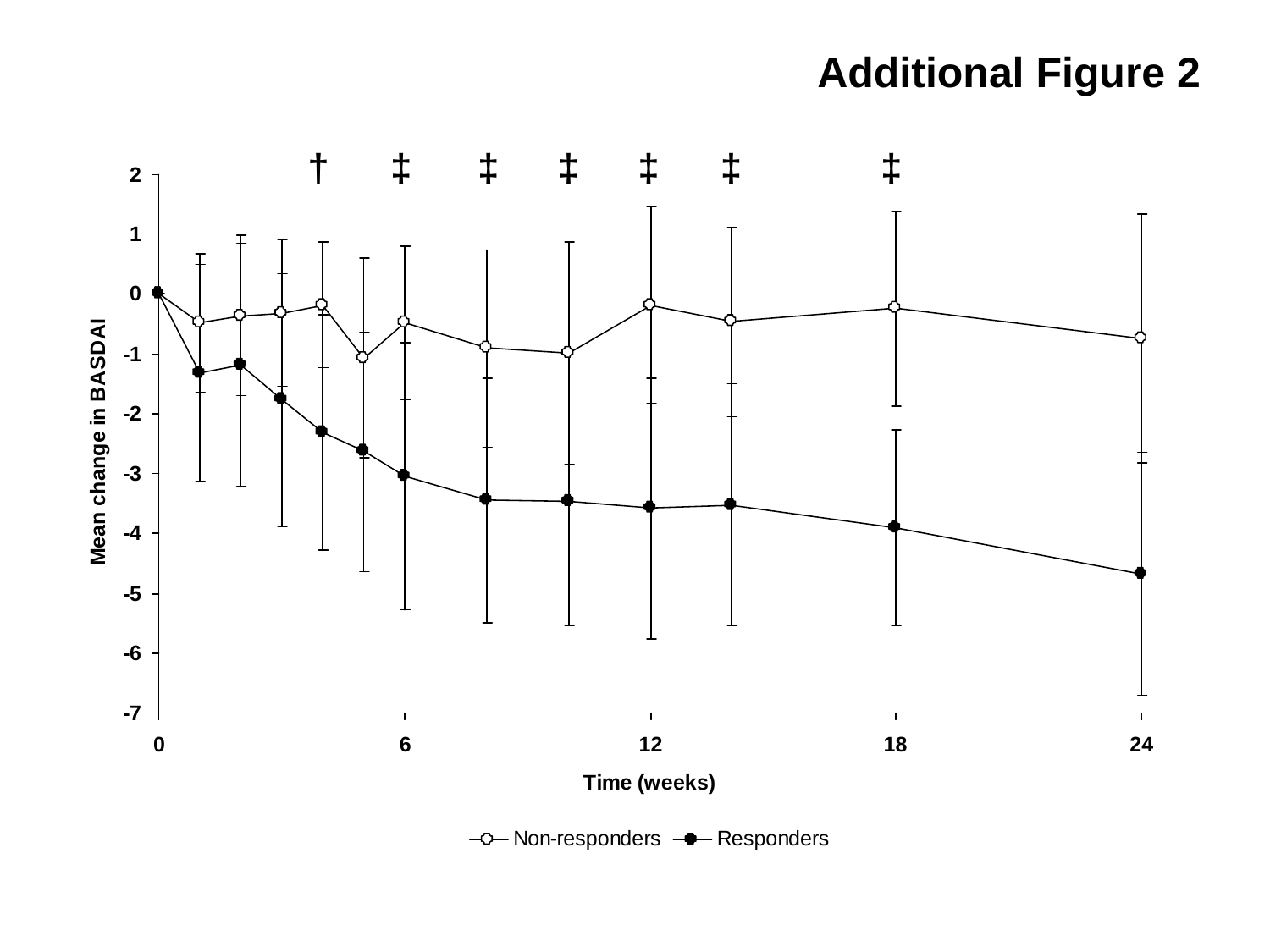

Additional Figure 2
†
‡
‡
‡
‡
‡
‡

Supplement: Additional file 2 — Figure S2. Changes in BASDAI scores for treatment responders (filled circles; n = 12) and nonresponders (open circles; n = 14) receiving infliximab alone or infliximab + methotrexate treatment. †P < 0.05. ‡P < 0.01. [file ar3350-S2.PPT]
